# Microstructure, Wear Resistance and Oxidation Behavior of Ni-Ti-Si Coatings Fabricated on Ti6Al4V by Laser Cladding

**DOI:** 10.3390/ma10111248

**Published:** 2017-10-30

**Authors:** Qiaoqiao Zhuang, Peilei Zhang, Mingchuan Li, Hua Yan, Zhishui Yu, Qinghua Lu

**Affiliations:** 1 School of Materials Engineering, Shanghai University of Engineering Science, Shanghai 201620, China; zh80836971@163.com (Q.Z.); 15317538065@163.com (M.L.); yanhua@foxmail.com (H.Y.); yu_zhishui@163.com (Z.Y.); luqh@sues.edu.cn (Q.L.); 2 Shanghai Collaborative Innovation Center of Laser Advanced Manufacturing Technology, Shanghai 201620, China

**Keywords:** Ni-Ti-Si composite coatings, laser cladding, wear resistance, high-temperature oxidation resistance

## Abstract

The Ni-Ti-Si composite coatings were successfully fabricated on Ti6Al4V by laser cladding. The microstructure were studied by SEM (scanning electron microscopy) and EDS (energy dispersive spectrometer). It has been found that Ti_2_Ni and Ti_5_Si_3_ phases exist in all coatings, and some samples have TiSi_2_ phases. Moreover, due to the existence of these phases, coatings presented relatively higher microhardness than that of the substrate (826 HV (Vickers hardness)) and the microhardness value of coating 3 is about twice larger than that of the substrate. During the dry sliding friction and wear test, due to the distribution of the relatively ductile phase of Ti_2_Ni and reinforcement phases of Ti_5_Si_3_ and TiSi_2_, the coatings performed good wear resistance. The oxidation process contains two stages: the rapid oxidation and slow oxidation by high temperature oxidation test at 800 °C for 50 h. Meanwhile, the value of the oxidation weight gain of the substrate is approximately three times larger than that of the coating 4. During the oxidation process, the oxidation film formed on the coating is mainly consisted of TiO_2_, Al_2_O_3_ and SiO_2_. Phases Ti_2_Ni, Ti_5_Si_3_, TiSi_2_ and TiSi were still found and it could be responsible for the improvement in oxidation resistance of the coatings by laser cladding.

## 1. Introduction

Titanium and its alloys are extensively applied in aviation manufacture, medical fields, marine industries and other industries due to their low density, high strength, excellent corrosion resistance and easy formation [1,2,3]. However, low hardness, poor wear and oxidation resistance severely restrict their further application fields [4,5]. To circumvent these problems, many technologies have been applied to improve the wear and corrosion resistance of titanium alloys to prolong their service time. Among them, the laser cladding has received more and more attention [6,7,8,9]. Huang et al. [10] fabricated TiVCrAlSi high entropy alloy coatings with little cracks and pores by laser cladding consisted of (Ti, V)_5_Si_3_ and a BCC (body-centered cubic) solid solution. Because of the combination of (Ti, V)_5_Si_3_ phase and ductile BCC solid matrix, the wear resistance in abrasive and adhesive wear coatings was improved significantly compared with the substrate. Weng et al. [11] fabricated metal matrix composites on Ti6Al4V titanium alloy by laser cladding. Because of the reinforced phases of CoTi, CoTi_2_, NiTi, TiC, TiB_2_, TiB, Cr_7_C_3_ and Ti_5_Si_3_, the coatings possessed high hardness and exhibited excellent wear resistance. The values of hardness of coatings were enhanced by 3–4 times compared with the substrate and the wear mass loss of coatings was approximately 0.083 of substrate. Dong et al. [12] obtained TiC reinforced Ti-Ni-Si intermetallic composite coating by laser cladding on TA15 titanium alloy. It can be seen that the wear resistance was particularly improved and the coating showed insensitive to the test load due to the uniform distribution of TiC in coating.

Many scholars have also been devoted to investigating the laser cladding coatings to enhance the oxidation resistance of titanium alloys at elevated temperature. Liu et al. [13] prepared the TiN/Ti_3_Al intermetallic composite coatings on Ti6Al4V substrate by laser cladding. The results of the isothermal oxidation test carried out at 600 °C showed that the value of relative oxidation resistance of laser cladding coatings was approximately six times bigger than that of the titanium alloy. This change may be attributed to the formation of TiN and Ti_3_Al disputing the diffusion of O atom. During the oxidation process, the affinity of Al and O is stronger than that of Ti and O [14] and the Al_2_O_3_ oxide would be preferentially formed on the composite coatings. Therefore, cladding coatings performed excellent oxidation resistance at high temperature. Liu et al. [14] investigated high temperature oxidation resistance of Ti_5_Si_3_/γ/TiSi composite coatings on γ-TiAl intermetallic alloy using NiCr-Si powders as precursor material. The conclusion drawn was that the coating consisting of NiCr-40%Si powders showed better high temperature oxidation resistance at 1000 °C for 50 h. Due to the brittle and loose TiO_2_, the oxidation products mainly consisted of SiO_2_ and Al_2_O_3_, which impeded the diffusion of O and improved the oxidation resistance of the cladding coating. Lv et al. [15] reported a study on the oxidation behaviors of TiNi/Ti_2_Ni composite coatings with TaC addition prepared on Ti6Al4V by laser cladding. After the oxidation test, the oxides could be found on the surface of coatings without TaC were TiO_2_, NiO, Cr_2_O_3_, Al_2_O_3_ and SiO_2_. While TaC was added, Ta_2_O_5_ and TaC were also found. Moreover, with the increase of TaC, the oxidation rates of coatings were decreasing. The oxidation resistance of coatings was remarkably improved at high temperatures.

There is very limited research on the wear resistance and high temperature oxidation resistance of Ni-Ti-Si laser cladding coatings on the titanium alloy. In this paper, the Ni-Ti-Si coating was synthesized on the Ti6Al4V titanium alloy by laser cladding with different ratios of Ti and Si powders. The chemical composition, microstructure, microhardness was investigated in sequence. Wear resistance mechanisms and oxidation behaviors at high temperature were also involved.

## 2. Materials and Methods

In this study, the substrate was Ti6Al4V titanium alloy with the dimensions of 50 mm × 50 mm × 5 mm. The chemical composite of the substrates is listed in Table 1. 

**Figure 1:**
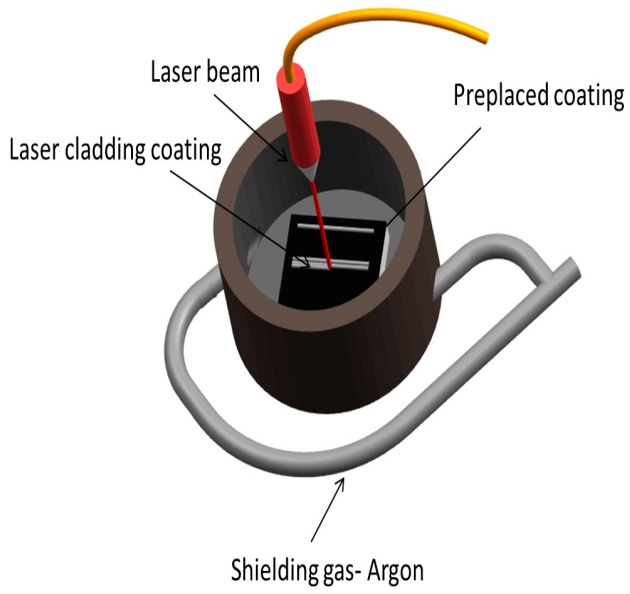
The schematic diagram of the shielded chamber.

Before laser cladding, the cladding surface was abraded and then cleaned ultrasonically with alcohol. Powders (≥99.5% purity) used as precursor materials have an average particle size ranging from 50–75 μm. Next, the powders were mixed in a ball mill for 3 h. The composition of cladding powders is given in Table 2. The mixed powders were pre-placed onto the substrate surface with the thickness of 1.0 mm. The powders were mixed with a binder (5% cellulose acetate + diacetone alcohol solution). The preplaced coating was dried for half an hour at 150 °C. Laser cladding was conducted in the argon shielded chamber of a 5 kW fiber laser material processing system (IPG YLS-5000, IPG Photonics, Boston, MA, USA) and the spot diameter is 5.0 mm. Figure 1 was the schematic diagram of the shielded chamber. The parameters of laser cladding are shown in Table 2. After laser cladding, the samples of the same coating were divided into four groups: Group 1 was for the XRD (X-ray Diffraction) test; Group 2 was for the microstructural analysis and microhardness test; Group 3 was for the wear test with an overlap ratio of 30%; and Group 4 was for the oxidation test.

The samples of Group 1 were cut and polished with abrasive paper of #150. Then, the constitution of the coating was identified by a PANalytical X’Pert Pro X-ray diffraction (XRD) (X’Pert Pro, PANalytical, Almelo, The Netherlands) with Cu Kα radiation (λ = 0.154060 nm). The samples of Group 2 were successively ground with abrasive paper of #150, #400, #800, #1200 and then polished with diamond paste. After this, the specimens were etched in a mixture solution (with the volume of HF:HNO_3_:H_2_O = 7:43:50) for 3–5 s. Microstructures and chemical compositions of the coatings were analyzed by a Hitachi S-3400N scanning electron microscope (SEM) (S-3400N, Hitachi, Tokyo, Japan) and a GENESIS EDAX energy dispersive spectrometer (EDS) (GENESIS, EDAX, San Diego, CA, USA) Finally, the microhardness along the depth direction of the specimen was detected by HXD-1000 Vickers (HXD-1000, Taiming Optical Instrument Co. Ltd., Shanghai, China ) with a load of 200 N and load-dwell time of 15 s. 

**Figure 2:**
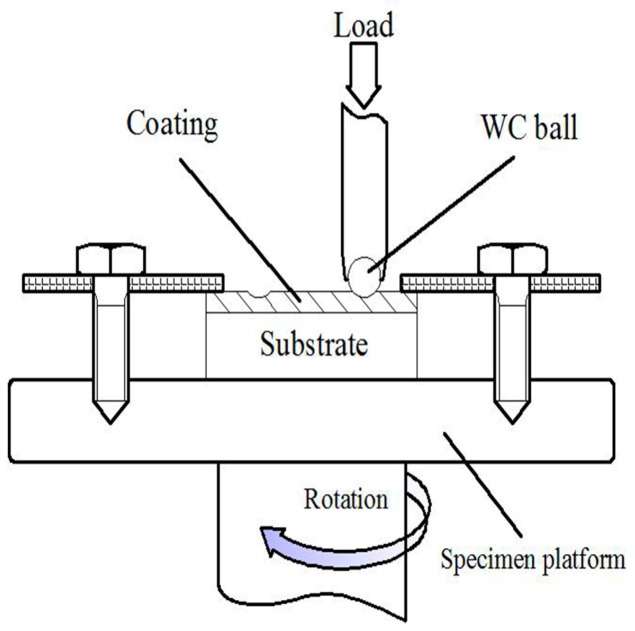
The schematic diagram of the wear test.

The dry sliding friction test for Group 3 was conducted on a CETR-UMT Multi-Specimen Test System (UMT-3, Bruker, Karlsruhe, Baden-Württemberg, Germany) at room temperature with an applied load of 150 N for 60 min. The schematic diagram of the wear test was presented in Figure 2. The adoptive rotation speed was 100 r/min and the friction radius was 3 mm. The topographies of wear surface were observed also by SEM. 

The samples of Group 4 were cut into plates with a size of 5 mm × 3 mm × 0.8 mm for the oxidation test. The surfaces of the specimen were ground by abrasive papers of #150, #400, #800, #1200 to make sure the surface smooth. The isothermal oxidation test was conducted in a muffle furnace at 800 °C for 50 h. The specimens were brought out once an hour to weigh the mass by an analytical balance with the accuracy of 0.1 mg.

## 3. Results and Discussion

### 3.1. XRD Results and Microstructures

**Figure 3:**
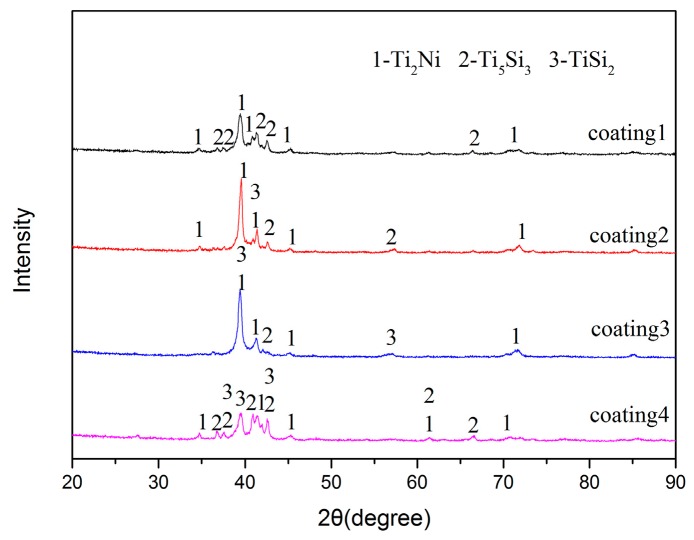
XRD patterns of the coatings.

**Figure 4:**
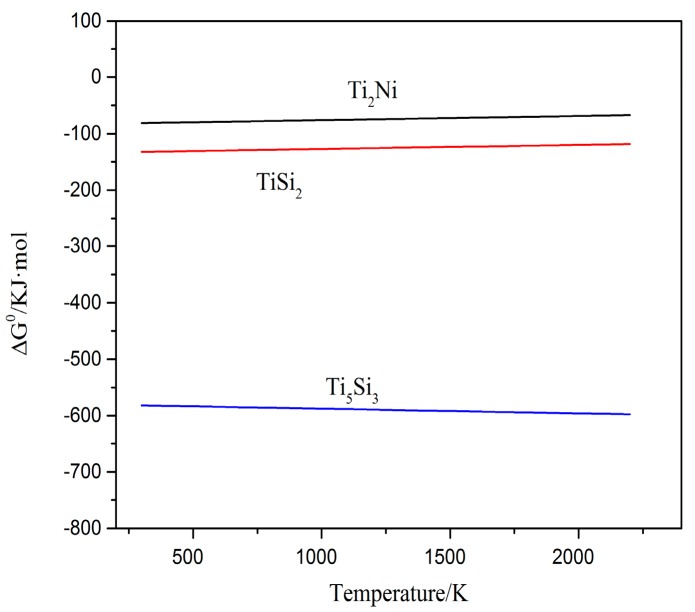
The Gibbs free energy (ΔG^0^/kJ·mol) of the formation of Ni-Ti-Si compounds.

The XRD patterns of the laser cladding coatings with different contents are shown in Figure 3. According to the Figure 3, it can be seen that the coatings are mainly composed of Ti_2_Ni, TiSi_2_ and Ti_5_Si_3_ phases. In general, laser cladding is a rapid melting and nonequilibrium solidification process; hence, multi-phases coexist and some diffraction peaks overlap, even diverging from the equilibrium positions in cladding coatings. With the content of Ti decreasing, the phases of the coatings change correspondingly. The diffraction peaks of coating1 are in accordance with JCPDS cards (No. 01-072-0442 and No. 00-018-0898 for Ti_2_Ni, No. 03-065-3597 for Ti_5_Si_3_). It can demonstrate that the coating 1 is primarily constituted of Ti_2_Ni and Ti_5_Si_3_ phases. The remaining three coatings have the same constitutional phases, which are Ti_2_Ni, TiSi_2_ and Ti_5_Si_3_. According to the data of Ref. [16], the values of Gibbs free energy (ΔG^0^) for the formation of Ti_2_Ni, TiSi_2_ and Ti_5_Si_3_ phases were theoretically calculated (shown in Figure 4). Based on the data in Figure 4, all of the ΔG^0^ are negative, which demonstrated that all of the phases can be formed spontaneously during the laser cladding. It can be seen that the Ti-Si compounds have more negative standard free energy. Moreover, the reach between Ti and Si is more stable than that of Ti and Ni. Ti and Si would tend to react with each other in the region rich in Ti and Si content. With the Si content increasing, the TiSi_2_ appeared.

**Figure 5:**
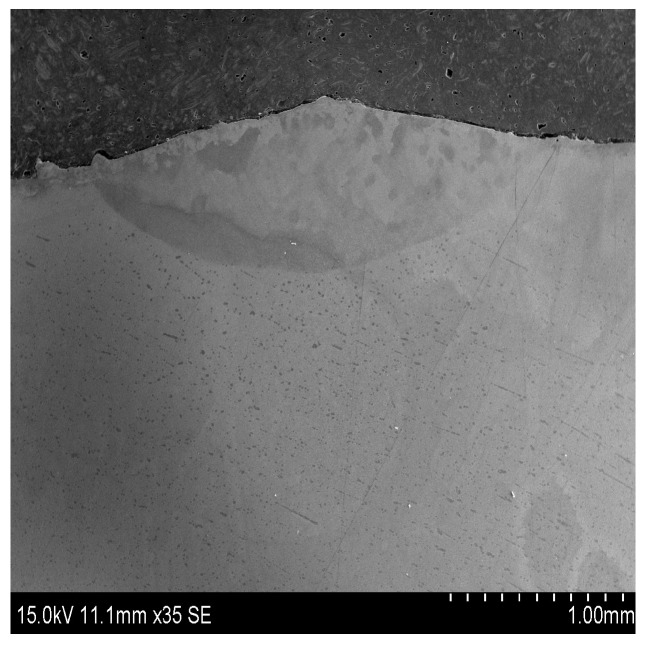
Macroscopic morphology in the cross section of coating.

Figure 5 indicates the macroscopic morphology in the cross section of coating. It can be found that the coating is metallurgically bonded well to the substrate and possesses dense microstructure without any microcracks and pores. The maximum thickness of the cross sectional profile of the coating is about 750 μm.

**Figure 6:**
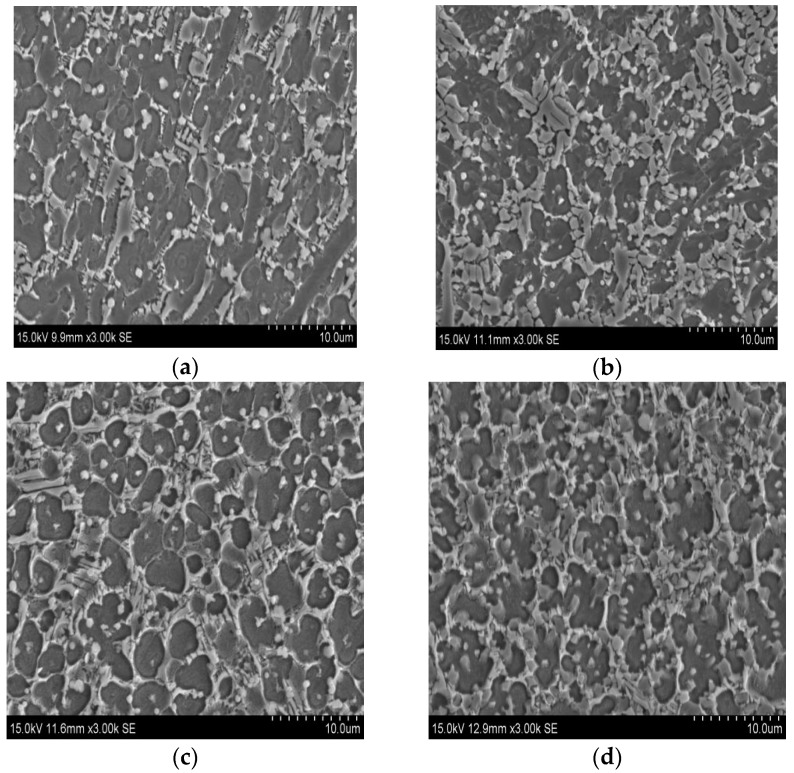
Microstructural characteristics of (**a**) coating 1; (**b**) coating 2; (**c**) coating 3; (**d**) coating 4.

**Figure 7:**
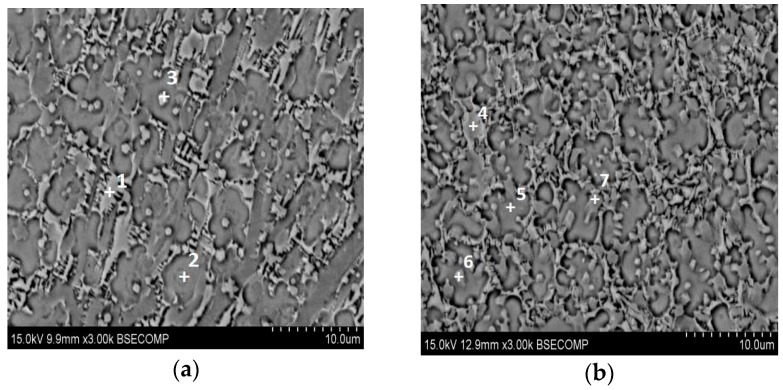
Microstructures of phases in coatings: (**a**) coating 1; (**b**) coating 4.

From Figure 6a, it can be seen that the coating is constituted of big black blocky phase, gray dendrites and a handful of white particles. With the decrease of Ti content, the number of the white particles is reduced, accompanied by the appearance of the greyish particles. To identify the chemical constitutions of these phases in coatings, EDS was used and the results are listed in Table 3. Because the laser cladding is a transient process, some elements in the substrate may be diluted into the coatings, such as Ti, Al and V. For coating 1 (Figure 7a), based on the data of phases 1 and 3, their atomic ratios of Ti and Ni are close to 2:1, respectively. Combined with the XRD patterns, the two phases can be identified as the Ti_2_Ni phase. Phase 2 is rich in Ti and Si whose atomic radios are approximately 5:3. Apart from the XRD results, phase 2 is supposed to Ti_5_Si_3_ reinforcement phase. The compositions of phases 4 and 6 are similar to that of phase 1 and 3, so phases 4 and 6 can be identified as Ti_2_Ni phase. Phase 5 is rich in Ti and Si, which is the same as the constitution of phase 2. On the basis of the result in Figure 3, it can be proved as Ti_5_Si_3_ phase. For phase 7, the total content of Ni, Ti, Al and V is nearly 35.84%, which is about half of the quantity of Si (64.16%). It reveals that phase 7 can be regarded as the TiSi_2_ phase. 

During the rapid solidification process, the intermetallic compound Ti_5_Si_3_ preferentially precipitates from the molten pool as the primary phase due to its relatively higher melting point (2403 K) and more negative free negative of formation. Accompanying the solidification of the primary Ti_5_Si_3_ phase, the amount of Ti and Si in the residual liquid is insufficient for the formation of Ti_5_Si_3_ phase, and, therefore, TiSi_2_ phase is formed. Hence, the residual liquid is rich in Ni. Finally, the Ti_2_Ni phase is formed by combining the Ti element, which may be from the substrate.

### 3.2. Microhardness and Wear Resistance

**Figure 8:**
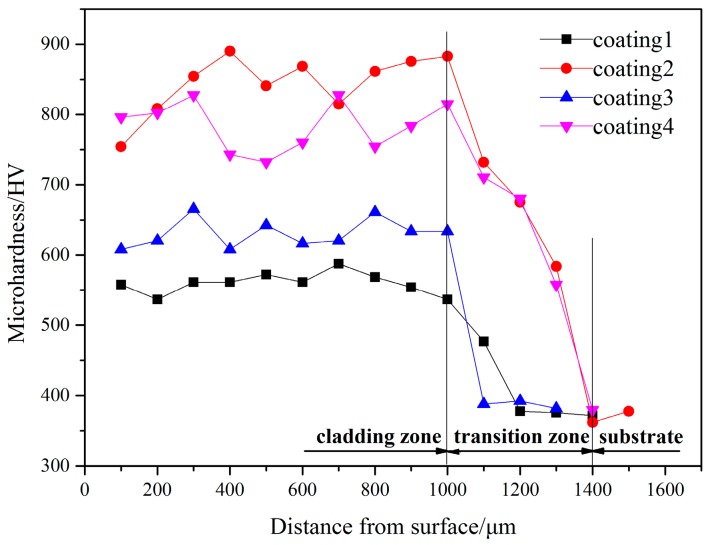
Microhardness of the laser cladding coatings along the depth.

Figure 8 shows the microhardness distribution of the cladding coatings along the depth. It can be seen that the microhardness of the coatings is distinctly different with the different contents of Ti. It may be attributed to the microstructural variations and the constitutions. Judging from the Figure 8, the results can be drawn that a transition zero existed between the substrate and cladding coatings and the value of microhardness of the coating near the substrate declines remarkedly. This is mainly owing to the coating diluted by the substrate. In addition, the microhardness of the zero is higher than the substrate (approximately 370 HV) due to the formation of the reinforced phases in the zero. The average microhardness of the coatings is about 560 HV, 826 HV, 628 HV and 765 HV, respectively. Compared with the coating 1, the microhardness of the remaining three coatings is all higher. This may be owing to the formation of the TiSi_2_ phase. Ti_5_Si_3_ and TiSi_2_ phases are both Ti-Si intermetallic silicides, which have a significant impact on the microhardness of coatings due to their characteristic advantages of high hardness, high melting points, and well oxidation resistance [17]. 

**Figure 9:**
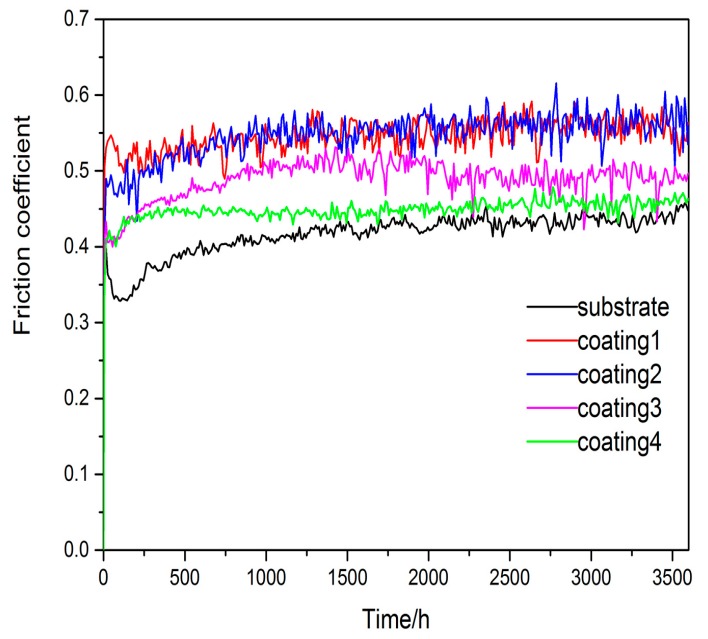
Friction coefficients of the substrate and laser cladding coatings at temperature.

**Figure 10:**
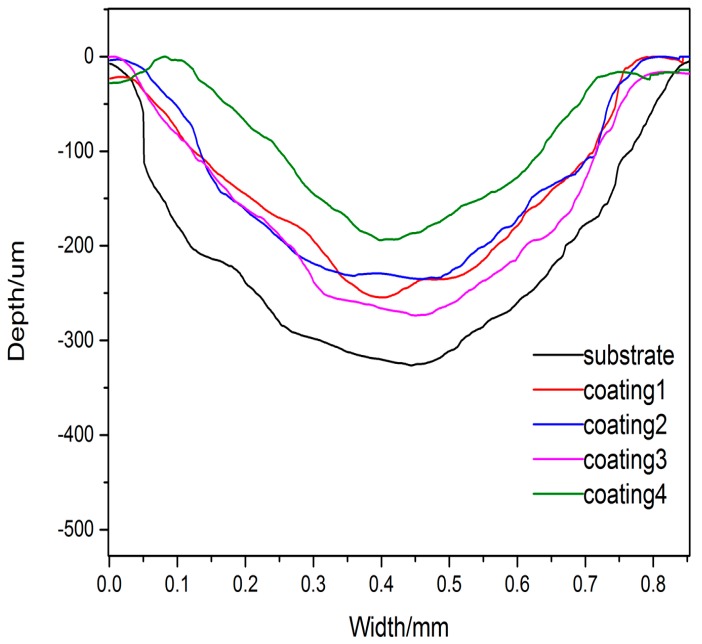
Profiles of the substrate and cladding coatings after friction and wear test.

The friction coefficients of the substrate and coatings against a WC (Tungsten Carbide) ball with the hardness of approximately 1600 HV are shown in Figure 9. The average friction coefficient of the substrate is 0.416 and it is slightly lower than the friction coefficients of coatings, which are 0.545, 0.546, 0.488 and 0.448, respectively. The friction coefficent of coating 4 fluctuates more smoothly than that of other coatings and substrate. It can be observed that with the decrease of the content of Ti, the average friction coefficients of the coatings reduce, simultaneously. Figure 10 shows the surface profiles across the wear scars of the substrate and the cladding coatings. The wear track width and the depth of all coatings reveal that the wear resistance of all coatings are well improved, especially coating 4.

**Figure 11:**
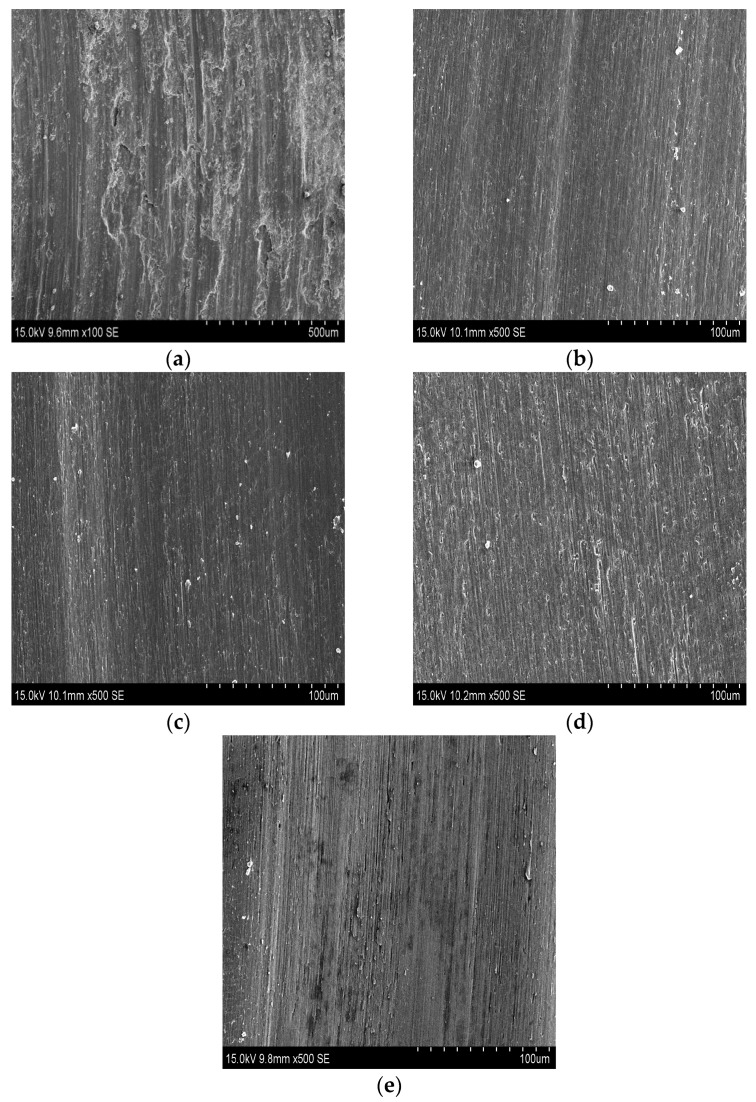
Worn surface morphologies of (**a**) the substrate; (**b**) coating 1; (**c**) coating 2; (**d**) coating 3; (**e**) coating 4.

To characterize the morphologies of the worn surfaces of the substrate and the coatings, SEM observation was carried out on the samples. As shown in Figure 11a, the substrate presents severe adhesive and abrasive wear due to the serious plastic deformation, deep plowing grooves and flaking debris. The substrate has low microhardness and hence the hard asperities of the WC counterbody are more likely to penetrate into the substrate, which results in the deep grooves. According to Figure 8, the cladding coatings all have comparatively higher microhardness than that of the substrate. Therefore, the wear mechanism of them is constituted of abrasive wear and the coatings exhibit a higher resistance to plastic deformation and scratching from the WC counterbody. The worn smooth surfaces of the coatings (Figure 11b–e) sufficiently demonstrate it. The hard silicide phases (Ti_5_Si_3_ and TiSi_2_) and relatively ductile phase (Ti_2_Ni) coexist in coatings. It is benefical for lessening the wear loss of materials. The Ti_5_Si_3_ phase plays an imperative role in abrasive and adhesive wear resistance due to its higher microhardness and large covalent-dominant atomic bonding. Furthermore, the Ti_2_Ni phase greatly combines the ductility and high microhardness, making a difference to support the hard silicide phase and defending the coating from brittle crack or fracture. Coatings exhibit outstanding wear resistance.

### 3.3. High Temperature Oxidation Resistance

**Figure 12:**
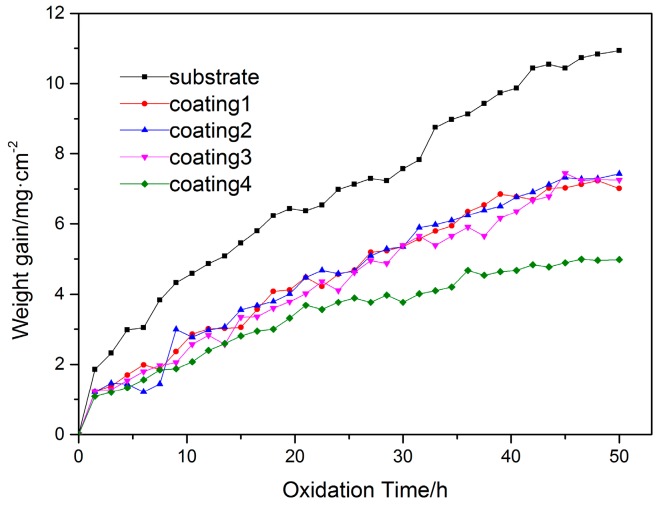
Oxidation weight gain curves of the substrate and cladding coatings at 800 °C.

Figure 12 presents the oxidation weight gain curves of the substrate and cladding coatings exposed at 800 °C for 50 h. Obviously, the weight of all the samples has a growing tendency with the elongation of oxidation time. In addition, the weight gain of coatings is clearly lower than that of the substrate at the same temperature during the oxidation. With the decrease of Ti content, the weight gain of coatings exhibits a regular change. For the coating with the Ti content between 30 at % and 50 at %, the weight gain fluctuates insignificantly. When the Ti content of the coating is 20 at %, the weight gain is decreased distinctly and about a half of the substrate. It can demonstrate that Ti content influences not only the phase constitutions of coatings but also the oxidation resistance.

**Figure 13:**
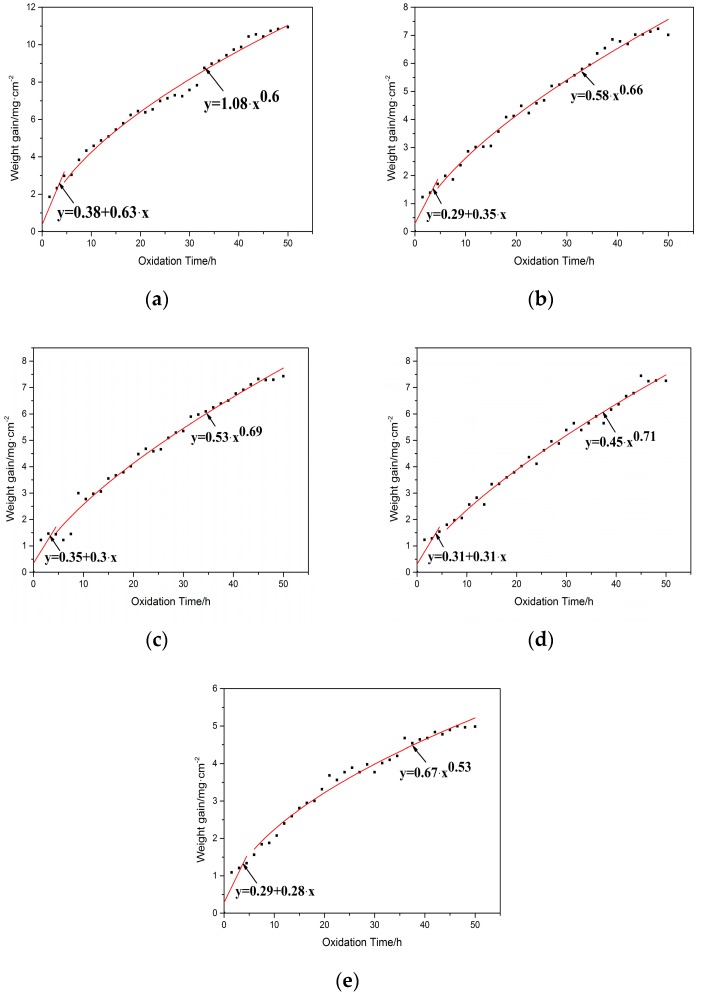
Oxidation weight gain fitted curves of the substrate and cladding coatings with different contents of Ti at 800 °C: (**a**) the substrate; (**b**) coating 1; (**c**) coating 2; (**d**) coating 3; (**e**) coating 4.

Based on the data acquired by high temperature oxidation (shown in Figure 12), the oxidation process for the substrate and coatings can be divided into two stages, which contains a sharp increase and relatively gentle growth in weight gain with the extension of time. During the initial stage, the speed of weight gain is quick because of the surface of samples is exposed in air without any protection. When the oxidation time is extended, the surface of the samples is surrounded by a layer of oxidation products that protect the samples are further oxidized. As a result, the fitted oxidation kinetics curves of them are different during different stages. In the first stage, the relationship between weight and oxidation time is fitted with a linear increase. The relative equation is listed as follows:

*y = a + bx,* (1)

where *y* is the weight gain and *x* represents the oxidation time. The values of *a* and *b* represent the intercept and the slope given in the table in Figure 13. The value of *b* is 0.379 mg/(cm^2^·h), 0.349 mg/(cm^2^·h), 0.345 mg/(cm^2^·h), 0.311 mg/(cm^2^·h), 0.289 mg/(cm^2^·h), respectively. It reveals that the oxidation rate of the coatings is lower than that of the substrate. Therefore, the oxidation resistance of the coatings can be improved compared with the substrate. In the second stage, the growth of oxides formed in the oxidation test can be described by reaction rate equation [18], and the equation is expressed as follows:

*y = mx^n^,* (2)

where *y* is the weight gain, *x* represents the oxidation time, *m* is the oxidation rate constant and *n* is the rate exponent. The value of *n* obtained for all samples is 0.587, 0.658, 0.685, 0.712 and 0.53, respectively, which is nearly close to 0.5. Hence, the oxidation kinetics curve of the samples after the first stage is in accordance with a parabolic law. 

**Figure 14:**
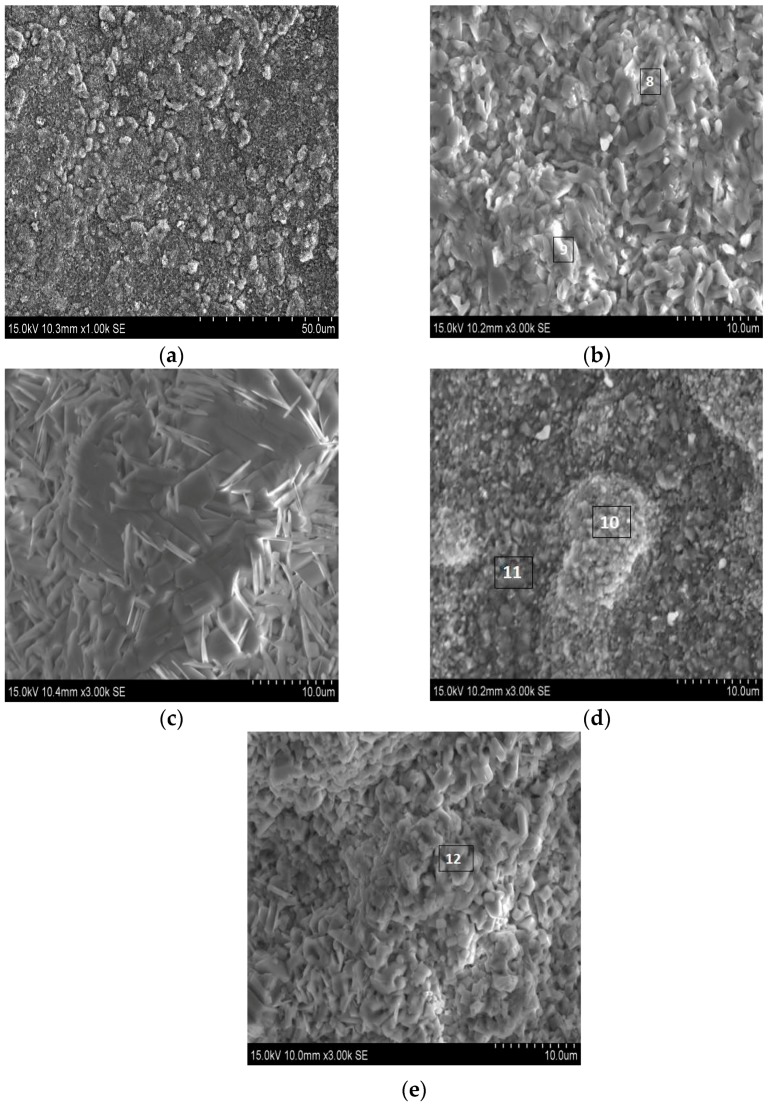
SEM morphologies of the substrate (**a**,**b**) and coating 4 (**c**–**e**) exposed at 800 °C for 50 h.

The SEM images and EDS analysis results of the substrate and coating 4 after high-temperature oxidation test for 50 h are shown in Figure 14 and Table 4. The oxidized surface morphology of the substrate shown in Figure 14a,b, Figure 14c,d, and Figure 14e reveal the oxidized surface morphology of coating 4. Based on the element content listed in Table 4, it can be drawn that the chemical constitution of the oxidized surface of the substrate only embraces Ti, Al and O, namely the titanium oxide and aluminum oxide may be formed on the surface of the substrate during the oxidation process. Regions 8 and 9 all show relatively high content of Ti, which indicates that the amount of titanium oxide is larger. The residual elements in the substrate are not detected. The low content of the residual elements in the substrate may be attributed to the phenomenon. According to the data in Table 4, the Ni, Ti, Si, Al and O elements are all detected in coating 4 after an oxidation test. That is to say, the oxides of coating 4 may contain four kinds. To identify the oxides in the samples, the XRD analysis is utilized.

**Figure 15:**
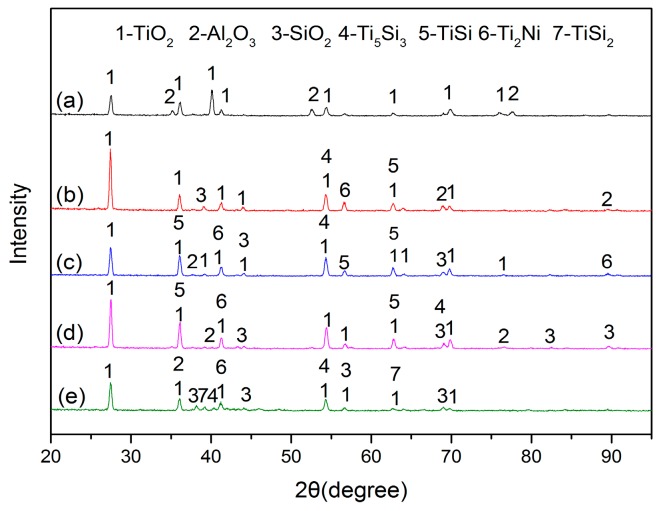
The XRD patterns of the specimens after oxidation test: (**a**) the substrate; (**b**) coating 1; (**c**) coating 2; (**d**) coating 3; (**e**) coating 4.

Figure 15 shows the XRD patterns of the oxidized films. Compared with oxides in the substrate that contain titanium oxides and aluminum oxide, the oxides of all coatings also involve the siilicon oxides. However, the oxides composed of other metal elements in material cannot be detected. It may be the low content of them that should be responsible for this phenomenon. Based on the data in Table 1, the chemical composition of the substrate is mainly composed of Ti and Al elements; hence, the oxides of the surface of the substrate are predominantly TiO_2_ and Al_2_O_3_. Because laser cladding is a rapid melting and rapid nonequilibrium solidification process, there is a small quantity of Al element diffused from the substrate to the coatings. Therefore, the oxide of coatings contains an Al_2_O_3_ phase. According to the XRD patterns, the phase constitutions of coatings exposed at 800 °C for 50 h are nearly the same. However, it is indisputable that there is no Ni oxide found despite the fact that the content of Ni in coatings is relatively high. It may be that the oxygen affinity between nickel and titanium is attributed to this phenomenon [19].

**Figure 16:**
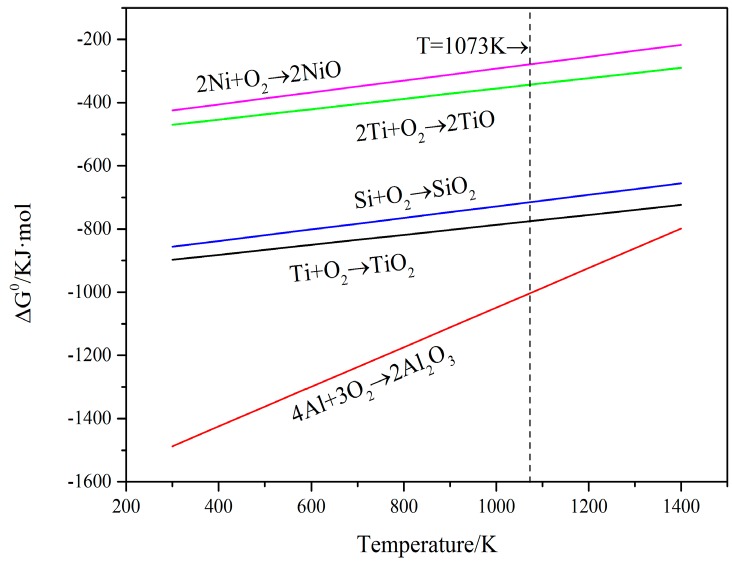
Variations of the standard free energy (ΔG^0^/kJ·mol) of formation of some oxides with temperature.

Generally speaking, the Ti element is comparatively easier to be oxidized than the Ni element at the same temperature. It can also be demonstrated by the results in Figure 16. However, Ti_2_Ni, Ti_5_Si_3_ and TiSi_2_ phases are simultaneously detected in coatings exposed at 800 °C for 50 h. During the oxidation process, only partial phases are oxidized. It can explain that the weight gain of coatings is lower than that of substrate, especially coating 4. The Ti-Si and Ti-Ni intermetallic compounds formed in coatings present better high-temperature oxidation resistance. In the coatings after oxidation tests, the TiSi phase is also detected. It may be ascribed to the reactions (3) and (4) [20]:

Ti_5_Si_3_ + Si → TiSi (3)

Ti_5_Si_3_ + O_2_ → TiO_2_ + TiSi (4)

Figure 16 offers the relationship between the standard Gibbs free energy (∆G^0^) of formation of the conceivable oxides with temperature. The thermodynamic data came from Ref. [17]. Apparently, the standard Gibbs free energy of the formation of Al_2_O_3_ oxide is more negative than others during the oxidation temperature (T = 1073 K). Under the circumstances, those with adequate contents of Ti, Al, Ni and Si, Al are apt to be preferentially oxidized at the test oxidation temperature [20]. Furthermore, the Al_2_O_3_ phase is more stable at the test temperature. The standard Gibbs free energy of formation of NiO is less negative than that of other oxides in the oxidation environment. It means that the NiO is relatively unstable and difficult to form. During the high temperature, Ti content may be oxidized to TiO and TiO_2_; however, only TiO_2_ is found in the XRD patterns. There are several reasons for this. Firstly, the standard Gibbs free energy of formation of TiO_2_ is more negative than that of TiO. In addition, TiO contains anion vacancy. It stands for the stability of TiO_2_ being relatively high and easier to be formed, and, secondly, the affinity energy of TiO_2_ formed by Ti and O is −54.99 eV, which is more negative than the affinity energy of TiO formed by Ti and O (−28.27 eV) [21]. With this reason, TiO_2_ in the practical oxidation process is generally detected compared with TiO while the monoxide is quickly oxidized to the dioxide [22]. It can be seen that the ∆G^0^ of TiO_2_ is just slightly more negative than that of SiO_2_ and a little more stable. Therefore, the sequential order of the oxides for Ti and Si may be uncertain.

During the initial oxidation process of coatings, O atoms in the air contact the surface of coatings and a layer of oxides is formed with the extension of time. TiO_2_ is brittle and loose in the oxide film and it is easier to detach from the surface. Then, O atoms can diffuse from the channel and are oxided with elements inside. For coatings, there is a large amount of Ti_5_Si_3_, TiSi_2_ and Ti_2_Ni reinforcement phases. They can hinder the diffusion of O atoms and restrain the oxidation process. Coatings present excellent oxidation resistance. It can be proved by data from Figure 12 and Figure 13. Moreover, coating 4 shows the best oxidation resistance.

## 4. Conclusions

Ti-Si and Ti-Ni intermetallic compounds composite coatings were fabricated by laser cladding using Ni-Ti-Si powders on Ti6Al4V substrate. The effect of the compounds on microstructure, microhardness and tribological properties were investigated. Moreover, the high-temperature oxidation resistance of the substrate and cladding coatings was also tested at 800 °C for 50 h. The following conclusions can be drawn:

- Coatings constituted of Ti_5_Si_3_, Ti_2_Ni and TiSi_2_ phases were obtained by laser cladding with different contents of Ni, Ti and Si powders. Because of the distribution of the inherent high hardness of these phases, the coatings have a higher microhardness than the substrate. While the proportion of the Ti content is 40 at %, the coating has the highest microhardness, which is 826 HV.
- By means of combining the reinforcement phases of Ti_5_Si_3_ and TiSi_2_ with the relatively ductile phase of Ti_2_Ni, the coatings present good wear resistance. Coating 4 shows the better wear resistance.
- The oxidation process can be divided into two stages, the rapid and the slow oxidation sections. The oxidation rate of the coatings is lower than that of the substrate during the process. With the decrease of Ti content, the oxidation rate gradually reduces. Moreover, coating 4 has the lowest oxidation weight gain rate. It can demonstrate that coating 4 presents the best oxidation resistance at high temperature.
- In the oxidation process, the oxides of coatings are mainly TiO_2_, Al_2_O_3_ and SiO_2_ compared with oxides of the substrate, which is only TiO_2_, Al_2_O_3_. Furthermore, the Ti_5_Si_3_, Ti_2_Ni, TiSi and TiSi_2_ phases are also found in coatings. It can be ascribed to the good oxidation resistance of coatings.

## Figures and Tables

**Table 1:** Chemical composite of Ti6Al4V titanium alloy (wt %).

Al	V	Fe	Si	C	N	H	O	Ti
5.5–6.8	3.5–4.5	≤0.30	≤0.15	≤0.10	≤0.05	≤0.015	≤0.20	Bal.

**Table 2:** The chemical composite of cladding powders (at %) and processing parameter.

Coating	Ni	Ti	Si	Laser Power/KW	Scanning Speed/(mm/min)
1	35	50	15	1.5	1000
2	35	40	25		
3	35	30	35		
4	35	20	45		

**Table 3:** EDS analysis results of the phases in coating 1 and coating 4 (at %).

Phase	Ni	Ti	Si	Al	V
1	23.65	63.28	2.87	8.25	1.94
2	3.37	65.04	34.77	6.39	3.37
3	22.65	69.86	2.81	3.41	1.27
4	27.45	62.06	3.11	5.93	1.46
5	3.05	58.26	22.98	2.53	1.39
6	28.05	57.73	3.25	8.92	2.04
7	8.14	17.13	64.16	8.38	2.19

**Table 4:** EDS analysis results of the regions in substrate and coating 4 after oxidation test (at %).

Regions	Ni	Ti	Si	Al	O
8	-	34.59	-	0.83	64.58
9	-	27.03	-	17.86	55.1
10	1.55	25.12	0.92	22.89	49.48
11	0.65	30.38	1.56	4.36	63.06
12	0.86	18.85	8.22	4.01	68.06
